# Dual-Path Effect of Mortality Salience Induced by COVID-19 on Food Safety Behavior in China

**DOI:** 10.3390/ijerph19106100

**Published:** 2022-05-17

**Authors:** Ying Ma, Xiaodong Guo, Weihuan Su, Yongxiang Feng, Fang Han

**Affiliations:** School of Management, Wuhan University of Technology, Wuhan 430070, China; 291431@whut.edu.cn (Y.M.); guo_xiaodong@whut.edu.cn (X.G.); 304391@whut.edu.cn (Y.F.); 307474@whut.edu.cn (F.H.)

**Keywords:** mortality salience, COVID-19, self-protective motivation, prosocial motivation, self-construal, food safety behavior

## Abstract

During the pandemic, the mortality salience of COVID-19 has affected everyone. The public is extremely sensitive to food safety, especially cold chain food and imported food. This research is based on the terror management theory, protective motivation theory, and self-construal theory. It proposes an integrated dual-path framework to explore the different mechanisms that mortality salience has on food safety behavior. The result of three experiments verified our conjectures. First, mortality salience positively affects individuals’ food safety behavior. More importantly, we found the dual-path mechanism that underlies the effect, that is, the mediating of self-protective motivation and prosocial motivation. In addition, different self-construals make the confirmed effect clear. These findings provide implications for the government to protect public food safety and health.

## 1. Introduction

The COVID-19 pandemic is a disaster for the whole world [1]. Since its outbreak, the virus has spread rapidly and constantly mutated [2]. As of 13 April 2022, nearly 500 million people have been infected, and more than 6.18 million have died [3]. These numbers are still increasing. Under these circumstances, the public has been inevitably exposed to messages or situations related to death [4] and suffered multiple pressures and fears of death [5]. Influenced by this panic, the public is extremely sensitive to safety information and may take extra precautions beyond official recommendations [6]. When individuals hear that a type of food may have the virus, even if it has not been confirmed, the food is immediately boycotted by the public [1].

Amid the pandemic, sensitive behavior has been caused by fear or mortality salience, and terror management theory (TMT) provides a viable framework to explain this effect [1,7,8]. According to TMT, individuals devise different defense mechanisms to minimize their anxiety from mortality salience and gain a sense of value or symbolic immortality [9]. In previous studies, self-esteem and cultural worldview were recognized by most scholars [7,9,10]. Self-esteem focuses on the promotion and protection of self-worth [11], while cultural worldview pays attention to the protection of group welfare and norms [12]. In the context of COVID-19, these two mechanisms provide some explanations for the impact of mortality salience on food safety behavior. That is, for the promotion of self-worth and the protection of self-health, people will purchase safe food. For the sake of public interest and group health, they will boycott unsafe food and spread negative news about it widely, even if the news is not confirmed. 

The above food safety behavior reflects the dual attributes of food safety, that is, the personal attribute of paying attention to one’s own interests and the public attribute of serving others [13,14,15]. To our knowledge, previous literature has adopted different defense mechanisms to explain specific behaviors [16,17,18], but no research has explored the impact of mortality salience on behaviors with different attributes. Thus, it is necessary to reveal the influencing mechanism of mortality salience on food safety behavior with two approaches. Self-protection motivation and prosocial motivation provide a framework to explain this. Driven by the self-protective motivation, individuals are more concerned about their own safety and health to choose safe food [19]. Furthermore, influenced by prosocial motivation, people tend to help others, maintain public food safety, and make larger contributions to society [18,20]. 

When facing stress or the threat of death, individuals with different personality traits have different cognitive and behavioral responses [21]. Individuals with an independent self-construal tend to take risks and focus more on self-interest [22], while those with interdependent self-construals are more concerned with collective interests [23]. This difference in behavioral responses has been significant during the pandemic [24]. 

This paper has three aims, (i) to investigate whether the mortality salience of COVID-19 affects food safety behavior, (ii) to explore the dual-path mechanism that mortality salience exerts on food safety behavior, and (iii) to test the moderating role of self-construal on the dual-path effect. The study provides a novel insight to explore the relationship between mortality salience and food safety behavior amid the COVID-19 pandemic. More important, the results of this study provide solutions for governments to deal with food safety panic during the epidemic and mobilize the public to protect public food safety and health.

## 2. Literature Review and Hypothesis Development

### 2.1. Mortality Salience and Food Safety Behavior

According to terror management theory (TMT), humans, like all other forms of life, have a survival instinct. However, unlike other organisms, humans’ intellect makes them aware that “the curtain will fall” [25]. This conflict between the desire for immortality and awareness of mortality has the potential to cause thanato-phobia, death anxiety, and existential anxiety in some people [16,25,26,27]. These individuals will experience overwhelming insecurity, especially when stimulated by real death events [18]. 

The ongoing pandemic has inevitably exposed all humankind to the fear of death. As COVID-19 rapidly spreads, people’s lives are filled with information about the pandemic [4]. This, in turn, activates their awareness of mortality and further evokes adverse emotional outcomes such as fear, anxiety, depression, irritability, stigmatization, sadness, boredom, and a sense of isolation [7,28]. A survey of 515 Chinese residents during the COVID-19 pandemic showed that their anxiety and awareness of mortality were both high and that they felt powerless about the current situation and immediate future [29]. 

To cope with these negative cognition states, individuals develop an “anxiety buffering system” that helps protect them against existential fragility. TMT argues that this system mainly includes two defense mechanisms: self-esteem and cultural worldview [7,9,10]. Specifically, self-esteem emphasizes self-worth, which is acquired by choosing high-priced products [11]. It motivates people to enhance their physical health and prioritize growth-oriented goals [30], such as quantified self-behavior [7], sustainable consumption [31], and green consumption [1]. The cultural worldview refers to the belief that people are valuable and meaningful within their own culture [12]. It can result in strong pro-social behaviors [26], such as seeking out opportunities to assist others, making charitable donations [32], engaging in environmental protection, and encouraging corporate social responsibility [33].

Food safety behavior can be defined as “the action taken by individuals to protect themselves and others from unsafe food or services” [13,14,15]. According to De Boeck et al.’s [34] definition and measurement of food safety behavior, this behavior has two aspects. On the one hand, the social public attribute is considered. That is, food safety is a key area of focus in public health, and people need to be encouraged to participate in its collective pursuit [35,36]. On the other hand, the health and safety attribute is considered. This means that consumers must take action to avoid exposure to biological, chemical, and physical hazards due to the frequent occurrence of food safety issues such as microbial contamination, adulteration, and misuse of food additives [37,38], to protect their health and safety [39].

As noted earlier, mortality salience has been associated with prosocial behavior [34] and health-oriented behavior [40]. When influenced by the cultural worldview, individuals’ prosocial motivation is stimulated. They are more likely to participate in social governance of food safety actively and execute voluntary food safety activities to promote others’ health and welfare [41]. When influenced by self-esteem, individuals promote benefits to themselves over benefits to others [42], pay more attention to self-protection, and enhance their health orientation behavior. Motivated by self-protection, individuals increase their safe food consumption and intervene in food insecurity behavior.

Thus, we propose that:

**Hypothesis** **1** **(H1).** *Mortality salience induced by COVID-19 is positively related to food safety behavior.*

### 2.2. Mediating Role of Self-Protective Motivation

Protective motivation theory (PMT) holds that all human behaviors are derived from a biologically based instinct for self-protection [43]. When confronted with the threat of self-interest, they ignore or distort it to suppress its positive impact on the self [44]. This motivation and corresponding behaviors are regarded as self-protection [45,46]. In terror management theory, the effect of self-protection is more obvious [19]. People marshal various resources to protect themselves from mortality anxiety [11,16].

Given the doctrine of PMT and TMT, we argue that amid the pandemic, individuals’ motivation for self-protection mainly comes from their instinctive response to the threat of mortality from COVID-19. Furthermore, considering the need for self-protection, individuals are more inclined to food safety behaviors (such as paying attention to food safety risks and buying safe food). Specifically, individuals are more concerned with their self-interest and generally make decisions that benefit themselves and their families compared with others. Second, the fear of the threat of COVID-19 has intensified the focus on personal safety and health; thus, people are choosing to buy safe food. Finally, the fundamental attribute of safe food enables people to feel safer during the pandemic.

Taken together, the mortality awareness caused by COVID-19 enhances the self-preservation motivation—the desire to defend one’s life and health. During the pandemic, individuals are choosing more conservative ways to avoid risks and reduce harm for the sake of their own interests and safety. Their food safety behavior is primarily derived from self-protection.

Thus, we propose that:

**Hypothesis** **2** **(H2).** *The self-protective motivation mediates the link between the mortality salience of COVID-19 and food safety behavior.*

### 2.3. Mediating Role of Prosocial Motivation

Prosocial motivation refers to a desire to give, help, benefit, contribute, protect, and promote the welfare of others [47]. According to self-determination theory (SDT), prosocial motivation comes from external (desire for reward or to avoid punishment) and internal (values or self-esteem) motivations [48,49]. As McAdams et al. [50] and Erikson [51] explained, the awareness of death reinforces two motivations: the desire to make a lasting contribution and the desire to connect with others. As Koole et al. [52] noted, the desire to make a lasting contribution is referred to as a buffer against death anxiety by extending a person’s contributions into the future. The desire to connect with others is regarded as a buffer against death anxiety by linking a person’s actions with groups, organizations, and institutions [43].

Thus, when confronted with the threat of death from COVID-19, individuals are more likely to value connection with others and desire to make lasting, self-transcendent contributions [4,53,54]. Specifically, it can be explained from three aspects. For one thing, the mortality threat of COVID-19 leads individuals to take personal responsibility for promoting the welfare of other people and the next generation [18,20]. For another, the pandemic increased the collective public concern about health. It strengthened collective public values, which are the intrinsic motivation of prosocial behavior [49] and an important factor for individuals to participate in public food safety governance. In addition, food safety behavior reflects the public interest [35,38]. The act of ensuring food safety is a behavior that protects and promotes the welfare of others.

In sum, the mortality anxiety from COVID-19 reinforces prosocial motivation—a desire to protect the public interest and the welfare of others. During the pandemic, influenced by collectivist values, individuals are more active in ensuring food safety and promoting the food safety of the public.

Thus, we propose that:

**Hypothesis** **3** **(H3).** *The prosocial motivation mediates the link between the mortality salience of COVID-19 and food safety behavior.*

### 2.4. Moderating Role of Self-Construal 

Self-construal is considered to be the way in which individuals construe themselves [55,56], which reflects the idea of “how individuals see the relationship between self and others” [57]. Self-construal is a multidimensional self-concept. Individuals with different self-construals show differences in cognition and motivation and reflect different consumption preferences and behavior habits [58]. 

Substantial research has demonstrated that independence and interdependence are the main dimensions of self-construction. The independent self-construal is bounded, unitary, and stable. It attaches more importance to the independence and uniqueness of the individual in thought and emotion. People with independent self-construal highly value the satisfaction of their own needs and pay more attention to the symbolic meaning and unique value of goods [59], so they tend to buy high-value, healthy, and safe products [60]. Meanwhile, the interdependent self-construal is flexible and variable. It emphasizes connections to others, nature, and the external environment. Thus, individuals with high interdependent self-construal are concerned with public benefits when making decisions and are more inclined to buy green products [55,59,61].

In addition, individuals with different personality traits make different judgments about stress and threat [62]. Thus, we can infer that facing stress or the threat of death has two response mechanisms according to different self-construals [21]. When facing the threat of death, individuals with independent self-construal may put more emphasis on self-protection [21], so they will try to take measures to protect themselves from the threat exposure. However, interdependent self-constructors may be more committed to observing group norms, protecting public interests, and avoiding social losses. This response mechanism is even more significant during the COVID-19 pandemic. Individuals mainly show two behavioral responses: one highlights self-protection and is scared of being infected by other people, which leads to actions such as wearing a facemask [63], increasing green consumption [64], attaching importance to food safety, and quantified self behavior [7]. The other emphasizes the protection of others and is scared of infecting others [65], which leads to actions such as taking the nucleic acid testing and following the quarantine regulations. 

Taken together, the mortality salience from COVID-19 has stimulated the self-protective motivation of individuals with independent self-construal, so they choose to buy safer and healthier foods. For individuals with interdependent self-construal, the mortality salience arouses their prosocial motivation, so they actively protect public food safety.

Thus, the following hypotheses are proposed:

**Hypothesis** **4b** **(H4a).** *Independent self-construal plays a moderating role in the relationship between mortality salience of COVID-19 and self-protective motivation.*

**Hypothesis** **4b** **(H4b).** *Interdependent self-construal plays a moderating role in the relationship between mortality salience of COVID-19 and prosocial motivation.*

**Figure 1:**
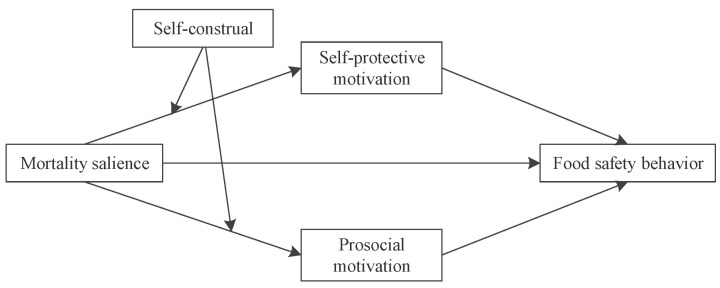
The conceptual framework.

The conceptual framework, see Figure 1.

## 3. Materials and Methods

### 3.1. Study Design

As discussed earlier, mortality salience triggers two behavioral motivations. One is the self-protective motivation, such as the need for self-health and safety [40]. The other is prosocial motivation, such as the concern for the welfare of others [43]. Influenced by these motivations, individuals are more likely to seek out and protect food safety. In addition, this study considers the role of self-construal in these relationships.

Taken together, we suggest that mortality salience of COVID-19 significantly affects food safety behavior (H1), which is mediated by self-protective motivation (H2) and prosocial motivation (H3); independent (vs. interdependent) self-construal moderate the link between the mortality salience of COVID-19 and self-protective (vs. prosocial) motivation (H4a/b). Three experiments were used to verify these hypotheses. In study 1, we adopted a one-way ANOVA to verify the impact of mortality salience of COVID-19 on food safety behavior. In study 2, we adopted a bootstrap method to test the mediating effect of self-protective (prosocial) motivation. In study 3, we adopted MANOVA to examine the moderating effect of self-construal.

### 3.2. Experimental Manipulation and Check

#### 3.2.1. Mortality Salience Manipulation

Narrative recall and video clips are often used to capture the features of different emotions [64]. Mortality salience was manipulated by edited videos (see Appendix A). According to Greenberg, Solomon, and Pyszczynski [9], subjects were assigned randomly to two experimental groups (mortality salience: high vs. low) and saw different videos. In the high mortality salience group, the video is predominantly black and gray, showing the news that the global death toll has exceeded 4 million during the COVID-19 pandemic and images of corpses lying in rows in some areas. In the low mortality salience group, the video has higher brightness, showing that since the outbreak of COVID-19, the public has been widely vaccinated, epidemic prevention and control have become normal, the economy is gradually recovering, and scenic spots have been opened to the public.

After watching the videos, subjects were asked to complete the scale of mortality salience which included 6 items (all items were scored on a 7-point Likert scale), which was adapted from Liu, Lv, and Tang’s [7] study. Items included, “The news about COVID-19 makes me feel anxious about my life”, “I feel terrified if I was infected COVID-19”, “I was worried that I would die because of COVID-19”, and so forth.

#### 3.2.2. Self-Construal Manipulation

Previous literature demonstrated that self-construal could be manipulated via the wording presented in a story [66,67]. Thus, we adopted a short story about a “tennis competition” to prime self-construal [68]. Subjects were randomly assigned to two experimental groups (self-construal: independent vs. interdependent). In the independent condition, the subject is an individual player. The reading material constantly emphasizes the importance of the self, such as “you are the center of the world”, “everyone fixes their eyes on you”, and “it is your chance” (see Appendix B). In contrast, in the interdependent condition, subjects are team players. The reading material constantly highlights the importance of the team and group, such as “your team are the center of the world”, “your team put a lot of effort into this match”, and “it is your team chance” (see Appendix B).

After the reading, subjects were asked to complete the scales of independent and interdependent self-construal (a total of 12 items were scored using a 7-point Likert scale) adapted from Aaker and Lee [69]. 

#### 3.2.3. Manipulation Check

The 80 subjects (34 women, 46 men), college students from a comprehensive university in the Wuhan area, verified our manipulations of mortality salience and self-construal. The results of one-way ANOVA analysis showed that participants in the high death group felt mortality more saliently than those in the low death group [M-hi = 4.52 vs. M-lo = 3.53, respectively; F (1, 78) = 43.576, *p* < 0.001]. Subjects in the independent group showed more themselves than those in the interdependent condition (M = 6.66 vs. M = 4.76, F (1, 78) = 42.418, *p* < 0.001). In contrast, subjects in the interdependent group showed more about others (M = 5.68 vs. 4.53, F (1, 78) = 83.927, *p* < 0.001). Thus, we confirmed the success of the manipulations of mortality salience and self-construal. 

### 3.3. Participants and Procedure

#### 3.3.1. Participants

In this study, three experiments were used to verify different hypotheses. To avoid adverse effects on the results due to the repeated participation of the same participant, the study recruited different participants to participate in the experiment for three days.

We recruited 94 [study 1, (54 male; 40 females)], 153 [study 2, (90 male; 63 females)], and 112 (64 male; 48 females) Chinese college students to participate in 3 experiments, respectively. Each person was paid RMB 5 for participating. 

Before the experiment, we needed to confirm that the subjects were physically and psychologically healthy, that is, with no physical or psychological diseases that could affect the experimental results. In terms of physiology, we inquired whether the subjects had visual or hearing impairments and if they had ever been infected with COVID-19. As for psychology, we inquired whether the subjects had ever had a mental illness and were preparing for or undergoing psychological treatment.

They all signed a voluntary experiment agreement and a privacy data protection agreement, and the experiment steps met the relevant requirements.

#### 3.3.2. Procedure

In study 1, we used the prepared videos to induce the different degrees of mortality salience (same as manipulation). Subjects were randomly assigned to two groups. After the video, participants were asked to complete the mortality salience scale and food safety behavior scale. We adopted the food safety behavior scale (8 items) revised by De Boeck et al. [34]. Sample items were: “buy safety food”, “follow food safety regulations”, “report the food incidents”, and “prevent others from buying”. Subjects used a 7-point Likert scale to answer the questions.

In study 2, we used the same procedure and video materials as in the manipulation to induce mortality salience. The subjects were assigned to two groups randomly. Next, they were asked to complete scales of mortality salience, food safety behavior, self-protective motivation, and prosocial motivation.

The self-protective motivation and prosocial motivation were used for mediation validation. To measure self-protective motivation, we adopted the scale (7 items) developed by Sedikides [44], Hepper, et al. [70] for self-protective motivation assessment. Sample items were: “I need to keep my health and safety”, “My family needs my protection”, and “When I fail, thinking it was due to luck”. We used 9 items from Grant and Wade-Benzoni [43] to assess prosocial motivation. Sample items were: “If I did, I would be appreciated”, “I have a moral obligation to do it”, and “I’m sure it’s important to do something good for society” (all items were rated on a 7-point Likert scale).

Study 3 adopted a 2 (mortality salience: high vs. low) × 2 (self-construal: independent vs. interdependent) between–subjects design. Specifically, the subjects were randomly allocated to four groups (high mortality—independent, high mortality—interdependent, low mortality—independent, low mortality—interdependent). The manipulation process of mortality salience and self-construal were the same as noted earlier. After that, subjects were asked to complete the scales of mortality salience, self-construal (noted earlier [69]), self-protection motivation, prosocial motivation, and food safety behavior.

## 4. Results

### 4.1. Study 1

First, we tested the reliability of the scales. The Cronbach’s alpha of mortality salient scale was 0.941, food safety behavior scale was 0.927. The results of the one-way ANOVA showed that subjects in the high perceived death group felt mortality more salient than those in the low perceived death group [M = 5.21 vs. 4.30, respectively; F (1, 92) = 50.709, *p* < 0.001]. Thus, the manipulation was confirmed to be a success. 

In this study, we used one-way ANOVA to test the main effect of mortality salience on food safety behavior. The likelihood of food safety behavior in the high mortality salience group was significantly higher than that in the low mortality salience group (M-high = 5.84, M-low = 4.78, F (1, 92) = 73.126, *p* < 0.001), supporting hypothesis 1.

The results of study 1 showed that compared with subjects exposed to low perceived death situations, subjects experiencing high mortality salience would be more likely to adopt food safety behaviors. This is also in keeping with the self-protection and self-actualization effect; for example, death-related thoughts can motivate people to enhance their physical health and prioritize growth-oriented goals [26]. Facing death, they have a greater desire to contribute to society and make more meaningful contributions to their own lives and careers [33].

### 4.2. Study 2

The Cronbach’s alpha of mortality salience scale was 0.872, the self-protective motivation scale was 0.962, the prosocial motivation scale was 0.975, and the food safety behavior scale was 0.928. The Cronbach’s alpha of these scales indicates strong internal consistency of the adopted scales. As expected, participants felt mortality more salient in the high perceived death (vs. low perceived death group) [M = 4.75 vs. 3.53, respectively; F (1, 150) = 159.060, *p* < 0.001], thereby confirming the manipulation of mortality salience was successful.

The result of one-way ANOVA showed significant differences in food safety behavior across the two groups [F (1, 150) = 52.790, *p* < 0.001]. The subjects were more inclined to adopt food safety behavior in high mortality salience (M = 5.91, SD = 0.62) than in the low mortality salience (M = 5.21, SD = 0.56). Thus, our results once again support H1.

Next, we considered whether high mortality salience positively affected self-protective motivation and prosocial motivation compared the other group. The ratings of self-protective motivation in the high mortality salience were higher than those in the low mortality salience [M-high = 5.18, SD = 0.68; M-low = 4.62, SD = 0.62; F (1, 150) = 28.256, *p* < 0.001]. And the result of prosocial motivation is similar (M-high = 5.88, SD = 0.97; M-low = 5.88, SD = 0.97; F (1, 150) = 28.108; *p* < 0.001). Therefore, the high mortality salience strengthened people’s self-protective motivation and prosocial motivation. In addition, the correlations that “self-protective motivation and food safety behavior” and “prosocial motivation and food safety behavior” were tested. The results indicated that both self-protective motivation and prosocial motivation have significant influence on food safety behavior (r^1^ = 0.602; *p* = 0.000; r^2^ = 0.507; *p* < 0.001).

**Figure 2:**
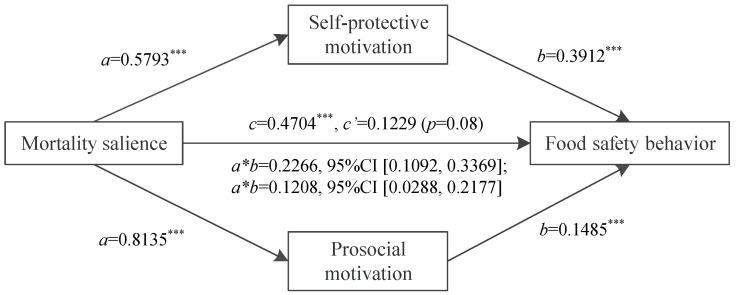
Mediating analysis of self-protective motivation and prosocial motivation. Note: “***” *p*-value < 0.001.

Then, we used bootstrap analysis to test the mediating role of self-protective motivation and prosocial motivation in the link between mortality salience and food safety behavior. According to Hayes [71], we adopted model 4 to run a mediational analysis. In the regression model, the dependent variable was food safety behavior, and the independent variables were mortality salience, self-protective motivation, and prosocial motivation. The effect of the mediators, self-protective motivation, and prosocial motivation, were significant (indirect effect = 0.227, 95% CI: [0.1092, 0.2239]; indirect path effect = 0.1208, 95% CI: [0.0288, 0.2177]). Moreover, the direct influence of the mortality salience on food safety behavior became nonsignificant when self-protective motivation and prosocial motivation were included in sequence (direct effect = 0.1229, 95% CI [−0.0149, 0.2607], *p* = 0.08), and the total effect of mortality salience on food safety behavior was significant (total effect = 0.4704, 95% CI [0.3413, 0.5994, *p* < 0.001]). Collectively, these results support hypotheses 2 and 3. Specifically, the self-protective motivation and prosocial motivation fully mediated the effect of mortality salience on food safety behavior. Figure 2 displays the complete path coefficients.

Taken together, the mediating effect of self-protection motivation and prosocial motivation showed a dual-path effect of mortality salience on food safety behavior. Specifically, for one thing, the threat of death from COVID-19 has aroused the desire of individuals to protect themselves and their families. They adopt healthier and safer lifestyles and therefore buy safe food. For another, facing a strong threat of death from COVID-19, individuals reflect on their misbehavior. At this point, individuals are stimulated to protect public food safety desires. 

### 4.3. Study 3

First, the manipulative effects of mortality salience and self-construal were tested. One-way ANOVA results showed that subjects felt mortality more salient in the high perceived death than low perceived death group (M-high = 6.03, SD-high = 1.17; M-low = 2.31, SD-low = 1.41; F (1, 112) = 240.60, *p* < 0.001, η^2^ = 0.68). The results of manipulation tests for self-construal showed that participants in the independent group reported a higher independent self-perspective than the interdependent group (M-in = 5.45 vs. M-inter = 4.97; F (1, 59) = 7.82, *p* < 0.01). Participants in the interdependent group reported a higher interdependent self-perspective than those in the independent group (M-inter = 5.44 vs. M-in = 4.99; F (1, 59) =6.89, *p* < 0.05), thereby confirming the manipulations of mortality salience and self-construal were successful.

**Figure 3:**
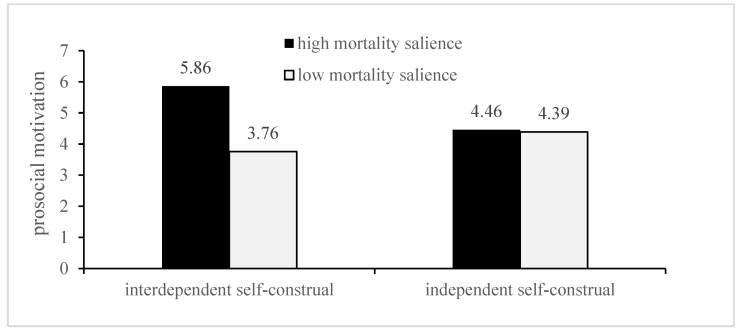
Moderating effect of self-construal type on the effect of mortality salience on prosocial motivation.

**Figure 4:**
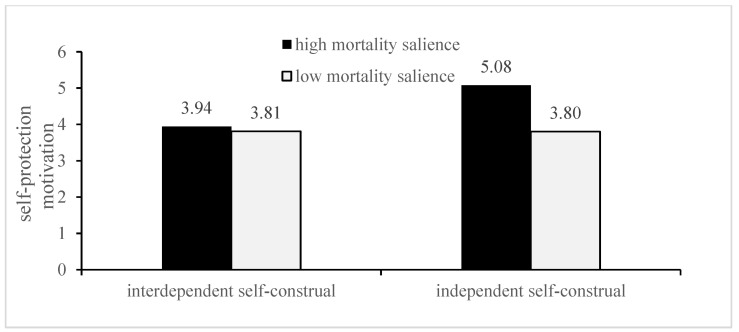
Moderating effect of self-construal type on the effect of mortality salience on self-protection motivation.

Furthermore, the intergroup comparison showed that, for the interdependent self-construal, the prosocial motivation of the high mortality salience group was significantly higher than the low group [M-high = 5.86, SD = 0.131; M-low = 3.76, SD = 0.258; F (1, 64) = 1743.650; *p* < 0.001]; for independent self-construal, there was no significant difference in prosocial motivation between the two groups [M-high = 4.46, SD = 0.37; M-low =4.39, SD = 0.419; F (1, 64) = 0.527, *p* = 0.471]. In addition, for independent self-construal individuals, the self-protective motivation in the high mortality salience group was significantly higher than the low group [M-high = 5.077, SD = 0.430 M-low = 3.801, SD = 0.0.591; F (1, 64) = 100.633, *p* < 0.001]; for interdependent self-construal, there was no significant difference between the two groups [M-high = 3.94, SD = 0.345; M-low = 3.809, SD = 0.2777; F (1, 64) = 2.890, *p* = 0.094]. Figure 3 and Figure 4 illustrate these results.

Finally, following a modulated mediation approach, we chose model 7 for data analysis [71]. In the regression model, the dependent variable was food safety behavior; the independent variables were mortality salience evoked by COVID-19 (high vs. low), self-construal, and motivation perspective (prosocial motivation vs. self-protective motivation). 

For the interdependent self-construal individuals, the mediating factor of prosocial motivation was significant (β = 0.488, SE = 0.087, 95% CI: [0.3224, 0.6720]), but the mediating factor of prosocial motivation was not significant for the independent self-construal individuals (β = −0.232, SE = 0.069, 95% CI: [−0.0281, 0.0651]). More, for the independent self-construal individuals, the mediating factor of self-protection motivation was significant (β = −0.232, SE = 0.069, 95% CI: [−0.3749, −0.1011]), but the mediating factor of self-protection motivation was not significant for the interdependent self-construal individuals (β = 0.008, SE = 0.012, 95% CI: [−0.1540, 0.0329]). Thus, mortality salience increased prosocial motivation for individuals with high interdependent self-construal, increasing food safety behavior. However, for those with high independent self-construal, mortality salience increased self-protection motivation, mediating the positive effect of mortality salience on food safety behavior. Collectively, these results support the notion that self-construal moderates the effect of mortality salience on prosocial motivation and self-protection motivation and then influences food safety behavior, thereby validating hypothesis 4a,b. The specific results are shown in Table 1 and Table 2.

## 5. Discussion

### 5.1. Theoretical Contributions

The current study adds to the theoretical contributions in three primary ways. Foremost, we contribute to the extant literature on the relationship between mortality salience and behaviors. Previous literature on TMT argued that individual behaviors are derived from defense against death anxiety and proposed different defense mechanisms to explain specific behaviors [16,17,18]. However, to our knowledge, some behaviors have both the personal attribute of satisfying oneself and the public attribute of serving others, such as green consumption behavior [64], intervention behavior, and food safety behavior of this study [35,38]. Thus, explaining the behavior with dual attributes from a single defense perspective may be incomplete. We fill this gap by introducing self-preservation motivation and prosocial motivation. This provides a basis for explaining the mortality salience and food safety behavior. This finding extends the research of Grant and Wade-Benzoni [43] and further explains the role of motivation in mortality salience and behavioral response.

Next, we extend the literature on TMT by establishing the dual mechanism of the mortality salience of COVID-19 on food safety behavior. Although research on TMT focused more attention on defense behaviors in the face of the threat of death, such as excessive consumption [16], quantified self-behavior [7], domestic brand preferences, and donation [33], no study has considered the impact of mortality salience on food safety behavior. During the pandemic, COVID-19 significantly impacted individuals’ food safety behavior. It manifests in two ways, choosing healthier food [72] and boycotting suspicious food [73]. At a deeper level, under the mortality threat of COVID-19, individuals may choose healthier food to protect themselves and intervene in unsafe behaviors for public safety, which reflects the mediating role of different motivations in mortality stimulus and behavioral response. Thus, we explain the impact of mortality salience on food safety behavior from the perspective of self-protection motivation and prosocial motivation. This finding is also helpful in analyzing green consumption behavior and intervention behavior from different motivational perspectives.

Furthermore, this research enriches the literature on behavior by exploring the boundary conditions (i.e., self-construal) on behavior. Although there are considerable discussions on the boundary conditions of mortality salience on behavior, including interest appeal [16], social distance [7], and social support [55], there are few arguments from the perspective of personality traits. Massive evidence has found that individuals will have different motivations and responses when facing different stimuli because of personal characteristics, cultural differences, and limitations of personal knowledge [64]. Thus, this study uses self-construal to explore how personality traits moderate the mortality salience and food safety behavior preference, that is, to explain how personality traits are consistent with behavioral motivations and food safety preferences. It further enriches the boundary research of the effect of mortality salience on behavior.

### 5.2. Practical Implications

This study provides some practical implications for food safety governance. 

Foremost, the fear of COVID-19 is obvious. Although it arouses individuals’ concern about food safety, it should not be encouraged as it may cause social disorder. During the pandemic, excessive panic and anxiety made the public sensitive to food safety, which also caused a lot of rumors about food (e.g., cherries spread the disease, the virus was found in strawberries, and a restaurant tested positive for the virus). Considerable rumors caused not only public panic but also huge losses to society and relevant enterprises. Thus, the government should take measures to buffer sensitive public emotions about food safety, detect and give early warning of food safety rumors, and avoid causing huge losses.

More importantly, these findings provide implications for government to achieve food safety co-governance. The low willingness of the public to participate in food safety governance, weak intention to intervene in food safety incidents, and ease of forgiving food safety misbehavior leads to frequent food safety incidents in China. Thus, the government should arouse the public’s willingness to participate in food safety governance. Specifically, on the one hand, publicizing the death threat of unsafe food through social media and press conferences motivates the public to protect themselves. On the other hand, we should pay more attention to the publicity of the public interest in food safety because, in China, the collective values are more prominent, and the interdependent personality is more obvious, so it is easy to stimulate the willingness of individuals to participate in food safety governance for the public interest.

In addition, current research also provides some implications for enterprises. Death information stimulates individuals’ strong demand for food safety self-protection, such as emphasizing the health and safety of food source, processing, storage, transportation, sales, catering, and other links to meet the public’s emotional needs. At the same time, it should be noted that during the sensitive period of mortality information (COIVD-19), food safety incidents are disastrous for enterprises, and much more attention should be paid to the supervision of food safety during the pandemic.

### 5.3. Limitations and Future Research

Our research has several limitations, which can be improved in the future. First, previous studies believed that self-construction varies under different cultural backgrounds [22]. For example, in the self-construal research, scholars directly identified samples from Asian countries (e.g., India, China, Japan, and Vietnam) as independent self-construal, and samples from European and American countries (e.g., the United States, Canada, and France) as interdependent self-construal [22,74,75]. Although our study manipulates self-construal based on Ma, Yang, and Mourali [68], it does not consider cultural factors. Therefore, in future research, samples from different regions will be selected to make the study robust. 

Furthermore, although existing studies have defined food safety behavior [13,14,15], it involves a lot of aspects, such as food safety protection behavior, intervention behavior, and consumption behavior, which provides opportunities for future specific food safety behavior research. 

Last, in recent years, the mortality salience of COVID-19 has attracted a lot of attention from different studies, but they have different views on the mortality salience effect. This indicates that the effect of mortality salience is also influenced by other factors, such as policy, culture, and region. Therefore, the influence of other factors on the effect of mortality salience can be explored in future studies.

## 6. Conclusions

This study explored the impact of the mortality salience of COVID-19 on food safety behavior and the underlying dual-path mechanisms. This research has shown that the mortality salience of COVID-19 has a positive impact on food safety behavior, but it needs to be interpreted from different perspectives. Specifically, self-protection motivation and prosocial motivation explain the dual-path effect of mortality salience on food safety behavior. In addition, self-construal moderated the dual-path effect of mortality salience and food safety behavior. That is, for individuals with independent self-construal, mortality salience enhanced the self-protective motivation. For interdependent self-construal, mortality salience increased prosocial motivation. Both have a positive impact on food safety behavior.

These findings not only contribute to the existing literature but also suggest that the government should adopt effective measures to relieve anxiety about food safety and COVID-19 to stimulate the public’s willingness to participate in food safety governance. In addition, it also provides a warning for food enterprises to enforce strict supervision and safe production.

## Figures and Tables

**Table 1:** Mediating role of prosocial motivation in moderating variables.

Type of Effect	Mediating Variable	Moderating Variable	Effect Size	*SE*	t	*p*	95% CI	
							LLCI	ULCI
Direct Effect	-	-	0.313	0.055	5.75	0.000	0.2094	0.4290
Mediation Effect	Prosocial motivation	Independent Self-construal	0.016	0.023	—	—	−0.0281	0.0651
		Interdependent Self-construal	0.488	0.087	—	—	0.3224	0.6720

**Table 2:** Mediating role of self-protective motivation in moderating variables.

Type of Effect	Mediating Variable	Moderating Variable	Effect Size	*SE*	t	*p*	95% CI	
							LLCI	ULCI
Direct Effect	-	-	0.383	0.069	5.526	0.000	0.2458	0.5200
Mediation Effect	Self-protection motivation	Independent Self-construal	−0.232	0.069	—	—	−0.3749	−0.1011
		Interdependent Self-construal	0.008	0.012	—	—	−0.1540	0.0329

## Data Availability

The data that support the findings of this study are available from the corresponding author upon reasonable request.

## Appendix A. Experimental Material of Mortality Salience

Video 1, the high mortality salience:

https://video.weibo.com/show?fid=1034:4767536185802791

Video 2, the low mortality salience:

https://video.weibo.com/show?fid=1034:4767540518518925

## Appendix B. Experimental Material of Self-Construal

You [Your team] are playing in a tennis tournament and have made it to the finals. [You are representing your team in the finals.] It is 4:26 p.m., and the sun is beating down on you. You count the strings on your racquet and bounce the ball a few times. At this moment, you are the center of the world. [Your coach and teammates fix their eyes on you.] You think to yourself: “This is my [our] battle. This is my [our] chance. Whether I win or lose, I will prove my worth to myself [my team].
